# Etymologia: β-Lactamase

**DOI:** 10.3201/eid2209.ET2209

**Published:** 2016-09

**Keywords:** etymologia, β-Lactamase, enzymes, antimicrobial resistance, antimicrobial drugs, bacteria

## β-Lactamase [baʹtə lakʹtə-mās]

Enzymes that catalyze the cleavage of β-lactam rings in penicillins, cephalosporins, monobactams, and carbapenems were first described by Abraham and Chain in 1940. These enzymes confer resistance to β-lactam antibiotics on bacteria that produce them. β-lactamases (Figure) are ancient, theorized to have evolved 1–2 billion years ago, but the emergence and spread of penicillin-resistant staphylococci in hospitals in the 1950s showed how penicillin use could select producers from a population of nonproducers. “Lactam” is a portmanteau of “**lact**one” (from the Latin *lactis*, “milk,” since lactic acid was isolated from soured milk) and “**am**ide.” The “β” refers to the nitrogen’s position on the second carbon in the ring. The suffix “-ase,” indicating an enzyme, is derived from “diastase” (from the Greek *diastasis*, “separation”), the first enzyme discovered in 1833 by Payen and Persoz.

**Figure:**
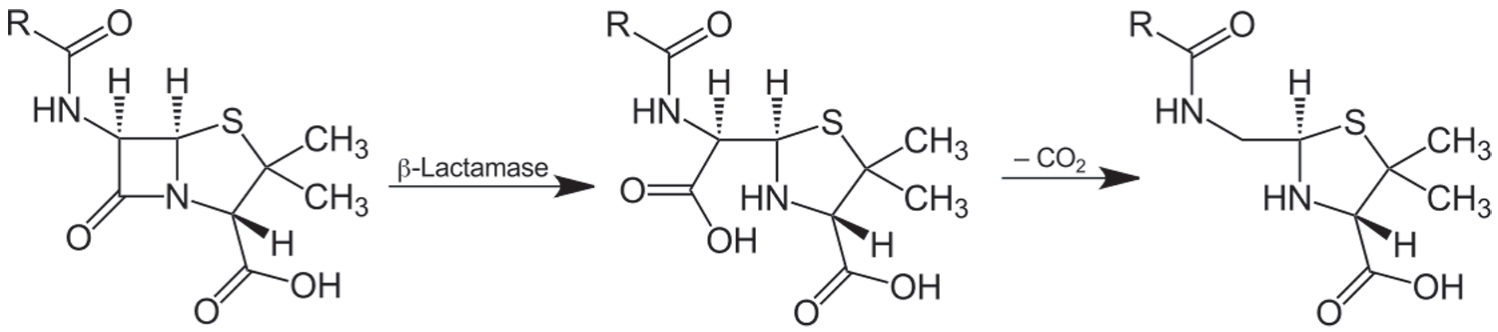
Action of ß-lactamase and decarboxylation of the beta-lactam ring. By Jü - Own work, Public Domain, https://commons.wikimedia.org/w/index.php?curid=11204303
